# Investigating the electronic structure of high explosives with X-ray Raman spectroscopy

**DOI:** 10.1038/s41598-022-24066-z

**Published:** 2022-11-14

**Authors:** Oscar A. Paredes-Mellone, Michael H. Nielsen, John Vinson, Konmeng Moua, K. Dean Skoien, Dimosthenis Sokaras, Trevor M. Willey

**Affiliations:** 1 SLAC National Accelerator Laboratory, 2575 Sand Hill Rd, Menlo Park, CA 94025 USA; 2 Lawrence Livermore National Laboratory, 7000 East Avenue, Livermore, CA 94551 USA; 3 Material Measurement Laboratory, National Institute of Standards and Technology, Gaithersburg, MD 20899 USA

**Keywords:** Chemistry, Physical chemistry, Physics, Chemical physics

## Abstract

We investigate the sensitivity and potential of a synergistic experiment-theory X-ray Raman spectroscopy (XRS) methodology on revealing and following the static and dynamic electronic structure of high explosive molecular materials. We show that advanced *ab-initio* theoretical calculations accounting for the core-hole effect based on the Bethe-Salpeter Equation (BSE) approximation are critical for accurately predicting the shape and the energy position of the spectral features of C and N core-level spectra. Moreover, the incident X-ray dose typical XRS experiments require can induce, in certain unstable structures, a prominent radiation damage at room temperature. Upon developing a compatible cryostat module for enabling cryogenic temperatures ($\approx$ 10 K) we suppress the radiation damage and enable the acquisition of reliable experimental spectra in excellent agreement with the theory. Overall, we demonstrate the high sensitivity of the recently available state-of-the-art X-ray Raman spectroscopy capabilities in characterizing the electronic structure of high explosives. At the same time, the high accuracy of the theoretical approach may enable reliable identification of intermediate structures upon rapid chemical decomposition during detonation. Considering the increasing availability of X-ray free-electron lasers, such a combined experiment-theory approach paves the way for time-resolved dynamic studies of high explosives under detonation conditions.

## Introduction

High explosive materials, used in a variety of civilian and military applications, release energy extremely rapidly when detonated. A challenge to a fundamental understanding of detonation phenomena is our inability to directly experimentally interrogate these rapid chemical kinetics within the bulk of these solid molecular materials. Particularly for dynamic detonation environments, theoretical and computational investigations have preceded experimental results by many years due to the complexity of experiments interrogating chemistry during detonation. As a prime example, the unique insensitivity to heat and shock and long reaction zone in tri-amino, tri-nitro benzene (TATB) may be correlated to the calculated rapid H$_2$O formation at the periphery of the TATB followed by formation of nitrogen heterocycles slowing detonation chemical reactions, but no experiments to date have verified this result^1^. Similarly, theoretical and computational chemical kinetics and decomposition mechanisms during detonation have been performed on various materials such as hexanitrohexaazaisowurtzitane (CL-20)^2^, 2,6-Diamino-3,5-dinitropyrazine-1-oxide (LLM-105)^3^, hexogen (RDX)^4^, octogen (HMX)^5^, and trinitrotoluene (TNT)^6^. Generally, the fundamental understanding of the underlying chemical kinetics may enable the targeted design of new explosives with desirable insensitivity and improved performance.

The element specificity of core-level spectroscopy can enable site-selective access to the local electronic structure providing information about neighboring atoms, coordination, and bonds. Application of core-level spectroscopies to high-explosives are scarce^7,8^. The intrinsic limitations of soft X-ray techniques regarding sample preparation and environment, along with the requirement for bulk sensitivity to investigate chemical decomposition and evolution during detonation conditions, pose major limitations to absorption spectroscopies for such organic species. X-ray Raman scattering (XRS) spectroscopy^9^, a core-level photon-in/photon-out hard X-ray technique, emerges as a powerful tool to address these fundamental questions. XRS provides bulk sensitivity and allows access to absorption edges of low Z elements with hard X-rays avoiding in this way usual constraints inherent to UV/soft X-ray spectroscopies. A built-in limitation of XRS is the low inelastic scattering cross-section, which requires both high-brilliance X-ray sources and large solid angle multi-crystal spectrometers to perform experiments in reasonable acquisition times for suitable high-concentrated systems. These aspects have delayed and limited the application of XRS.

In this work, we establish a requisite static experimental and computational framework to characterize the electronic structure of common high explosives. Three materials investigated here represent both canonical and unique properties among explosives. TATB, an incredibly stable and insensitive high explosive, consists of alternating nitro (NO$_2$) and amino (NH$_2$) moieties attached to a central six-membered carbon ring, while hexanitrostilbene (HNS), also a relatively stable material, contains NO$_2$ but lacks NH$_2$. These materials provide spectroscopic baselines for these two nitrogen groups that are often found in high explosives. CL-20, one of the most powerful manufactured explosives, is a cage structure, with saturated rather than aromatic carbon. By combining experimental and theoretical methods, we establish a synergistic framework to identify and distinguish un-detonated explosives from transient structures by unambiguously assigning spectral features to the molecular structure of the system. Ultimately, this systematic study of high-explosives upon static conditions sets the basis for future time-resolve dynamic experiments during detonation, by leveraging the increasing availability of X-ray free electron laser facilities around the globe^10^.

## Results and discussions

### Room temperature XRS measurements on TATB and HNS

**Figure 1:**
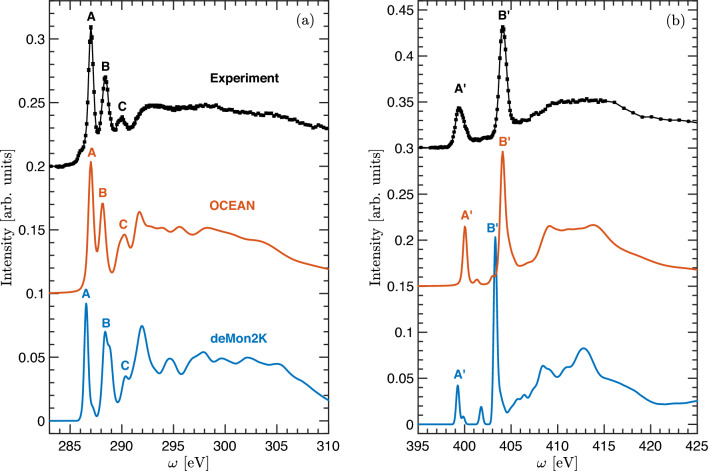
Experimental and theoretical 1s carbon (**a**) and nitrogen (**b**) X-ray Raman spectra of TATB.

The TATB carbon and nitrogen 1*s* XRS experimental spectra along with the corresponding ocean and deMon2K calculations are shown in Fig. 1.

The main features in the experimental carbon 1*s* spectrum are: a small pre-edge shoulder with an onset at 285.78 eV, two strong sharp peaks at 287.02 eV (A) and 288.40 eV (B), and a lower intensity peak observed at 290.02 eV (C). Transition energies are listed in Table 1. Most of these features are well captured by the calculations both in terms of energy positions and intensities, with the exception of the low-energy pre-edge shoulder. Another difference between experiment and calculations is observed for energies above $\approx$291 eV, where both codes predict slightly more pronounced fine-structure than experimentally observed, something quite common for DFT approaches. Similarly, on the nitrogen 1*s* spectrum we observe two marked peaks at 399.40 eV (A’) and 404.11 eV (B′) as shown in Fig. 1b.

The excellent agreement between calculations and experiments, particularly for the BSE-based approach, allows us to associate the spectral resonances to the molecular and electronic structure of the system. The ocean code calculates the excited state (photoelectron—core-hole pair) wavefunction iteratively taking into account the core-hole interaction explicitly within the BSE approximation^11^. Ultimately, this procedure provides the excited state 3D electron densities associated with the transitions.

**Table 1:** Transition energies of noticeable spectral features. Listed energy values correspond to peak maximum with the exception of TATB pre-edge where the onset value is given instead^12^. Note, only relative energies are calculated with ocean (see text), and peak A (B′) of TATB was used to align the carbon (nitrogen) K edge.

Molecule	Feature	Energy transfer [eV]	ocean energy [eV]	deMon2K energy [eV]
TATB	pre-edge	285.78±0.07	–	–
	A	287.02±0.02	287.02$^c\!\!\!$	286.56
	B	288.40±0.02	288.14	288.40
	C	290.02±0.03	290.25	290.04
	A’	399.40±0.02	400.04	399.26
	B′	404.11±0.02	404.11$^c\!\!\!$	403.30
HNS	A	285.01±0.03	284.93	284.69
	B	285.88±0.02	285.59	285.80
	C	288.28±0.02	287.93	287.51
	A’	399.7±0.1	–	–
	B′	404.02±0.02	403.45	403.08
CL-20	A	287.84±0.04	–	–
		286.88±0.03$^{a}$	–	–
		286.91±0.03$^{b}$	–	–
	D	$\approx$289$^{b}$	289.08	288.86
	B	291.61±0.08$^{a}$	–	–
		291.71±0.04$^{b}$	291.00	291.72

$$^{a}$$10 K - high dose.

$$^{b}$$10 K - low dose.

$^c\!\!\!$ocean requires a single calibration energy.

**Figure 2:**
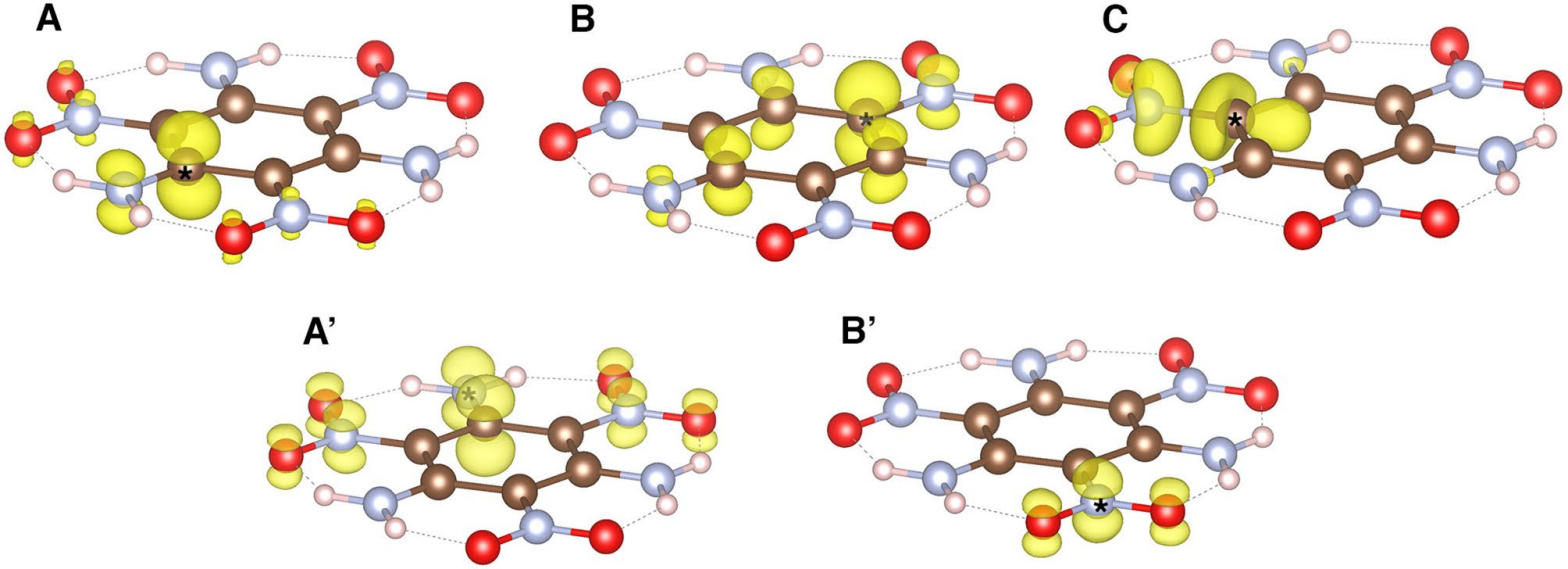
Excited state charge densities of carbon (top) and nitrogen (bottom) sites contributing to transitions as indicated in Fig. 1. Color code: Brown: carbon. Gray: nitrogen. Red: oxygen. White: hydrogen. Excited sites are indicated by an asterisk. Figures were made using VESTA^13^.

The excited state charge densities associated with the carbon and nitrogen spectral features are shown in Fig. 2.

These densities illustrate that the first (A) and second (B) peaks on the carbon 1*s* spectrum correspond to carbons bonded to NH$_2$ and NO$_2$ groups, respectively, and the probed states exhibit a strong *p*-like character. These results are in contrast with previous bond-assignments^7^ where, based on intramolecular charge transfer analysis, A and B were assigned to C-NO$_2$ and C-NH$_2$ moieties, respectively. The peak C observed at higher energies, has strong contribution from in-plane $\sigma$-like excitations from carbon sites bonded to NO$_2$ groups. The excited state electron densities associated to both peaks of the nitrogen 1*s* spectrum exhibit a dominant $\pi$-like character with nitrogen sites from NH$_2$ and NO$_2$ groups contributing to A′ and B′, respectively, though the former exhibits a higher degree of delocalization (see Fig. 2). Additional support to the energy positions found in this work for structures A (A′) and B (B′), and the corresponding site assignments, is provided by previous X-ray photoelectron spectroscopy (XPS) results on N 1*s* core-level binding energies for different compounds bearing NH$_x$ 399.5 ± 0.2 eV^14^, 400.4±0.1 eV^15^, 400.49±0.08 eV^16^ and NO$_y$ 404.2 eV^17^, 403.9 eV^17^, 403.7 eV^18^, 403.6^19^ groups.

**Figure 3:**
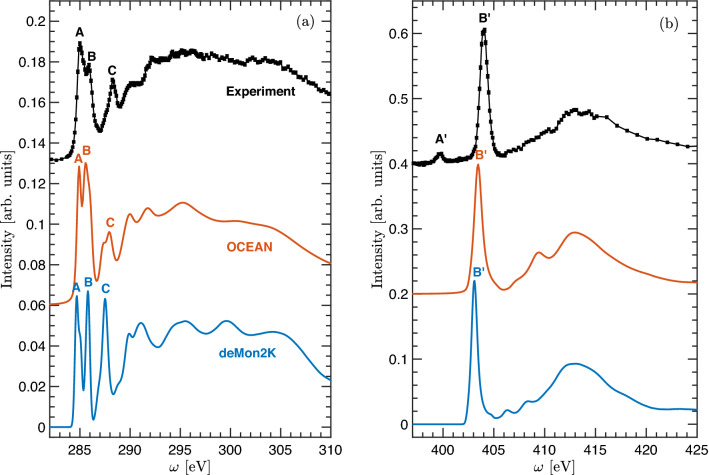
Experimental and theoretical 1s carbon (**a**) and nitrogen (**b**) X-ray Raman spectra of HNS.

**Figure 4:**
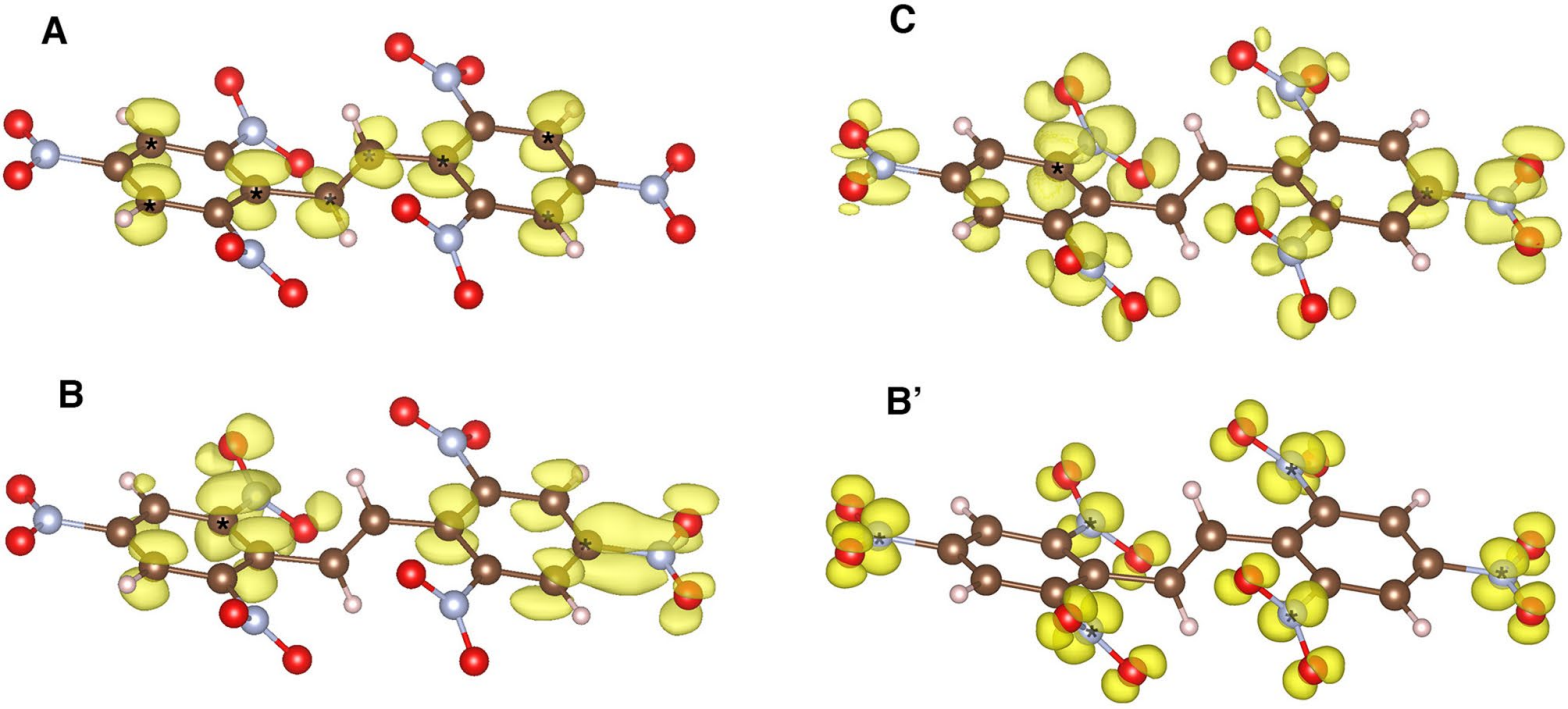
Excited state charge densities contributing to peaks in the carbon and nitrogen 1*s* spectra as indicated in Fig. 3. Atoms color code and labeling as in Fig. 2.

Experimental and calculated carbon and nitrogen 1*s* spectra of HNS are shown in Fig. 3a and b, respectively. The first peak observed in the experimental carbon 1*s* spectrum has a sharp maximum (A) at 285.01 eV and a marked high-energy shoulder (B) at 285.88 eV, followed by another sharp structure (C) at 288.28 eV. For the nitrogen edge, an intense sharp peak (B′) at 404.02 eV appears and a very weak pre-edge structure (A’) at 399.7 eV. The pristine HNS molecule constitutes of a single nitrogen species formed by six NO$_2$ groups. Therefore, the weak pre-edge structure represents a minimal contribution of contamination and/or early radiation damage. Besides this, the main observed features are nicely reproduced by both calculation approaches, although ocean predicts energy positions and intensities of the spectral features in a noticeably better agreement with the experiment. Electron charge densities associated with the relevant structures in the spectra are shown in Fig. 4. The first transitions contributing to peak A arise from *p*-like symmetry charge densities highly localized around the excited carbon sites. Those transitions come from C-H moieties and the carbons bridging between the two C6 rings. The major contribution in transitions at B comes from more delocalized states associated with carbons in the C-NO$_2$ groups exhibiting a $\pi$-like bonding between the CN sites. At feature C, higher energy transitions from C-NO$_2$ carbon moieties contribute to largely delocalized charge densities. All nitrogens contributing to B′ exhibit similar *p*-like symmetry charge densities localized around the atoms within the NO$_2$ groups.

### Room vs cryogenic temperature XRS measurements on CL-20

**Figure 5:**
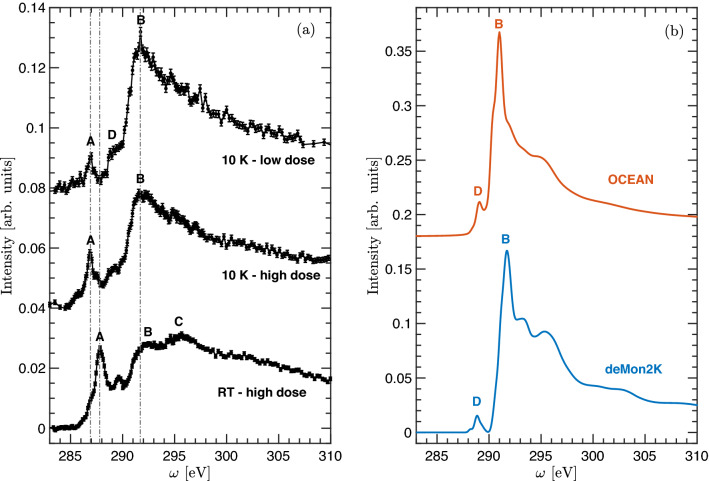
Experimental (**a**) and theoretical (**b**) 1*s* carbon X-ray Raman spectra of CL-20. Experimental spectra were taken for different radiation dose and temperature as indicated.

Carbon 1*s* spectra of CL-20 are shown in Fig. 5. The first set of measurements was performed at room temperature, same as for TATB and HNS with an acquisition time per individual spectrum of $\approx$ 260 s^20^. The bottom spectrum in Fig. 5a shows an intense sharp peak at 287.84 eV (A), followed by a marked feature at 289.6 eV and two prominent broad structures (B and C) at higher energies. The corresponding calculated ocean and deMon2K spectra are shown in Fig. 5b. The spectral features calculated by both codes are very consistent, predicting similar intensities, peak energy positions and overall spectral shapes. When compared to the measured RT CL-20 spectra striking differences are evident suggesting quick radiation damage from the intense incident X-ray beam. Radiation damage on high-explosives has been previously reported on X-ray diffraction experiments using synchrotron radiation^21^. Within the biochemistry and biology communities it is well known that such effects get suppressed when samples are kept at cryogenic temperatures^22,23^, therefore measuring radiation-sensitive high-explosive molecules under cryogenic conditions could allow the acquisition of reliable experimental spectra for the pristine molecular structures. Here, we designed a custom cryostat compatible with the geometry of the XRS experiments and enabled X-ray Raman experiments at cryogenic temperatures down to $\approx$ 10K. In this way, by measuring CL-20 at 10K, and for similar radiation dose as in room temperature measurements ($\approx 210$ s per spectrum), major spectral changes are observed in the carbon 1*s* spectrum. The first peak A shifts to lower energies and its intensity decreases. Additionally, the broad structure at higher energies becomes more intense and pronounced at 291.61 eV (B). After further reducing the radiation dose down to $\approx$ 55 s per spectrum, a similar trend is observed, the intensity at A diminishes even further with no apparent shift in energy and B evolves into a marked peak. Additionally, a pre-edge shoulder emerges around $\approx 289$ eV (D) which corresponds very well with theoretical spectra. The observed systematic changes in the spectral features with increasing radiation dose and temperature, along with the remarkable agreement between simulated and low-dose spectra measured at 10 K, are conclusive evidence that the appearance of peak A and the decrease in intensity at B are spectral fingerprints associated with radiation damage and do not represent features of the pristine molecular structure.

Theoretically, unimolecular decomposition pathways in high-explosives have been investigated by means of density functional theory^2,24^ and *ab initio* molecular dynamics under high-temperature conditions^25–27^. By assessing the high-sensitivity of X-ray Raman spectroscopy to the local electronic and molecular structures of high-explosives, and our recently developed capabilities for investigating radiation sensitive systems, this study set the basis for future systematic XRS experiments to investigate such decomposition mechanisms and shed light on the fundamental chemistry processes high-explosives undergo during initial decomposition steps. Performing high-*q* X-ray Raman experiments could also provide complementary/additional information for characterizing the electronic and molecular structure of these materials through the opening of strong contributions to the XRS spectra of non-dipole-like excitation channels.

## Conclusion

We have investigated high-explosives with X-ray Raman scattering spectroscopy. By combining experimental and advanced theoretical methods, excellent agreement between measurements and simulations is achieved. The BSE approximation has proven to be fundamental in accurately describing near-edge spectral features, relative intensities, and energy positions. For the case of TATB and HNS, the experiment-theory agreement allowed an unambiguous assignment of spectral features to specific moieties of the system. For systems with high sensitivity to radiation damage, we developed capabilities to perform X-ray Raman scattering experiments at cryogenic temperatures, which proved to suppress the damage induced by the intense incident X-ray beam. XRS measurements at cryogenic temperatures allowed reliable data acquisition of the pristine, undamaged system. These results constitute conclusive evidence of the suitability and sensitivity of XRS spectroscopy in identifying spectral fingerprints in high-explosives.

## Methods

### Sample preparation and handling

Samples were prepared by uniaxially pressing materials into small disks to minimize the energetic material in any given sample. For TATB and HNS these disks were 3 mm in diameter and had a weight of 10 mg. The CL-20 samples were pressed into 6 mm diameter, 40 mg disks to accommodate mounting within the cryostat, and to enable multiple fresh acquisition positions on a given sample. The room temperature samples were mounted using SEM adhesive tape. Cryostat samples were mounted in a custom-fabricated cassette to hold the samples in place without imposing any mechanical stress. All explosive samples were transported and handled under U.S. Department of Transportation (DOT) and Department of Energy (DOE) regulations.

### Experiment

The X-ray Raman scattering (XRS) spectroscopy experiments were performed at BL 15-2 at Stanford Synchrotron Radiation Lightsource (SSRL). The undulator radiation was monochromated using a liquid nitrogen cooled Si(311) double-crystal monochromator. Two Kirkpatrick-Baez (KB) mirrors focused the X-ray beam to a 50 $\times$ 150 $\mu m$ (H $\times$ V) size at the sample position with a photon flux of $3 \times 10^{12}$ photons/s to $4 \times 10^{12}$ photons/s. All samples were measured in reflection geometry (along the horizontal plane) using a grazing angle of $\approx$ $2^{\circ }$ to $3^{\circ }$ in order to optimize counting statistics and minimize incident beam areal power density. The XRS spectrometer at the BL 15-2 end-station constitutes of 40 diced Si(110) analyzer crystals and more details are given elsewhere^28^. The center of the spectrometer corresponds to a forward scattering angle of $\approx$40$^{\circ }$ with an angular opening of about 50$^{\circ }$. Measurements of the energy-loss spectra were performed in the so-called inverse geometry, *i.e.* the analyzed energy was kept fixed at $\omega _2 \approx$ 6.46 keV while varying the incident beam energy $\omega _1$. Hence, the XRS spectra are measured by scanning the energy transfer $\omega =\omega _1-\omega _2$ around each absorption edge. The total energy resolution at the operated Si(440) diffraction order and for the adopted grazing geometry and beam size was measured to be $\approx$ 0.33 eV via the full width at half maximum (FWHM) of the elastic peak. The angular range of the spectrometer captures momentum transfers between 0.7 a.u.$\le$ *q* $\le$ 2.9 a.u. For these *q* values and for the studied absorption edges C and N, the dipole approximation $q a < 1$ (*a* being the 1*s* core electron orbital radius) is fulfilled. Hence, the information accessible by XRS within this regime is equivalent to that of X-ray absorption spectroscopy. For each sample and absorption edge, several individual X-ray Raman spectra were collected across numerous fresh sample spots under the same experimental conditions. The measured intensity of each spectrum was normalized to the monitored intensity and used for extracting an averaged spectrum. Accurate energy-loss calibration was obtained by periodically measuring the elastic line. The variance in the elastic line energy position, as obtained by fitting a Gaussian profile, was $\le 0.01$ eV. To extract the core-electron contribution from the whole energy-loss spectrum, we performed a combined fitting procedure scheme similar to the one described elsewhere^29,30^. An exponential decay function was fitted for energy transfers below the 1*s* electron excitation onset to account for the high energy tail of the valence electron spectrum. Simultaneously, we approximate the high-energy tail of the whole energy-loss spectrum by the exponential decay function plus the atomic background as calculated with the FEFF code^31^. The latter was used to approximate the high-energy tail of the core-electron contribution. Finally, the fitted exponential function obtained out of this combined procedure was subtracted from the spectra to obtain the core electron contribution. Additional details are provided in the supplementary material.

### Calculations

Geometry optimizations and numerical frequency calculations were performed using the B3LYP exchange-correlation functional with triple-zeta basis functions with the ORCA 4.2.1 package^32^. For the optimized geometries, vibrational frequencies were calculated and no imaginary frequencies were obtained from the analysis. The theoretical X-ray spectra were calculated with two different approaches. First, a density functional theory (DFT) based formalism using Gaussian type orbitals as basis sets and the so-called transition potential approximation for determining the oscillator strengths was used as implemented in the deMon2K package^33^. Second, a plane-wave DFT formalism which takes into account excitonic effects explicitly within the Bethe-Salpeter equation (BSE) approach was used as implemented in the ocean code^34,35^. Unlike deMon2K which calculates only X-ray absorption spectra, the ocean code can calculate X-ray Raman spectra by accounting for the finite momentum transfer, which can enable the interpretation of high-*q* XRS experiments. Details of the X-ray spectra calculations are given in the supplementary material. The theoretical spectra were broadened with an energy-dependent Lorentzian and constant Gaussian line shapes as done elsewhere^36,37^. The former accounts for core-hole lifetime and the latter for instrumental resolution. For the TATB carbon (nitrogen) edge, we applied a Lorentzian broadening of 0.1 eV for $\omega <286$ eV ($\omega <403$ eV), which then linearly increased up to 3 eV between 286 eV $\le \omega \le 310$ eV (403 eV $\le \omega \le 420$ eV). Then, the spectra were broadened with a Gaussian lineshape of FWHM=0.33 eV. A similar broadening was applied for plotting the HNS and CL-20 theoretical spectra.

## Supplementary Information

Supplementary Information.

## Data Availability

The datasets used and/or analysed during the current study available from the corresponding author on reasonable request.
