# Supplementary material for: Investigating the electronic structure of high explosives with X-ray Raman spectroscopy
Source: Sci Rep. 2022 Nov 14;12:19460. doi: 10.1038/s41598-022-24066-z (PMC9663711; doi:10.1038/s41598-022-24066-z)
Supplement: Supplementary file 1 — Supplementary Information. [file 41598_2022_24066_MOESM1_ESM.pdf]

Supplementary material

Calculation details

deMon2k

Directionally averaged polarization vector x-ray absorption spectra were calculated within the transition potential approximation as implemented in the deMon2K package[1] . The BLYP exchange correlation potentials and a general GGA optimized double zeta valence polarization basis set was employed[2] . For the core-excited atom site, we used an IGLO-III basis set which was augmented with a set of diffuse basis functions[3,4]. For all other atoms of the same type as the absorbing site, effective core potentials for the 1s shell were employed to avoid orbital mixing. Calculated transition potential energies for each site were brought to an absolute energy scale by calculating the corresponding energy position of the first core-excited state as given by the difference, in total energy (including exchange and correlation), between the system with a full core-hole 1s level (with an electron at the lowest unoccupied state) and the ground state of the system[5]. The above basis set was then used to perform ground state and full 1s core-hole calculations for each absorbing site. Then, the XAS spectrum is obtained by averaging over the energy-corrected contribution of each absorbing site in the system. Additionally, the resulting energies were further corrected by a rigid shift of the total spectra to account for relativistic effects[6].

OCEAN

X-ray Raman spectra for finite momentum transfer were calculated with the OCEAN code[7,8]. Orbitals were obtained from the plane-wave DFT package QUANTUM ESPRESSO[9]. Norm-conserving pseudopotentials generated with the ONCVPSP code[10–12], modified for OCEAN, were used. We adopted the following approach to model molecules with OCEAN, which uses periodic boundary conditions. We centered the optimized coordinates of a single molecule inside a rectangular box. Convergence was checked so that increasing the dimensions of the box produce negligible changes in the near-edge features of the spectra. An energy cutoff of 104 Ry was set for the plane wave-basis. For all molecules, the isotropic electronic contribution to the dielectric constant was set to 1.01. The cut-off radius for the evaluation of the local screening response was set to 9.5 a.u. For TATB, a k-point sampling of $2\times2\times6$, $2\times2\times4$ and $1\times1\times2$ was used for ground state, final state and screening wavefunctions, respectively. Similarly, for HNS the respective k-points were set to $2\times2\times3$, $1\times2\times2$ and $1\times1\times1$, and for CL-20 to $3\times3\times3$, $2\times2\times2$ and $1\times1\times1$. The box dimensions containing the molecule were approximately $24.4\times22.7\times10.9$ a.u.^{3} for TATB, $42.9\times29.7\times23.8$ a.u.^{3} for HNS and $23.8\times23.8\times23.8$ a.u.^{3} for CL-20. For calculating the spectra, a real-space sampling grid of $26\times24\times14$, $32\times24\times24$ and $28\times28\times28$ was used for TATB, HNS and CL-20, respectively. OCEAN can calculate relative core-shifts consistently between different systems by properly scaling the number of bands respect to one of the systems which is chosen as reference. Once a proper number of conduction bands N_C (screening bands N_S) has been obtained for the reference, a linear relation between the number of bands and the number of valence electrons n_V per unit cell is established $N_C = n_V + A_C V$ ($N_S = n_V + A_S V$), where V is the volume of the unit cell. The scaling factors A_C (A_S) were chosen such that the highest unoccupied state was approximately 45 eV (100 eV) above the lowest unoccupied molecular orbital for TATB. Then, this scaling constant is used to determine the proper number of bands in the other systems. Hence, all calculations can be brought to an absolute energy scale by aligning one of the calculated spectra to the experiment and direct comparison of the core-shifts for all systems can be performed. For our calculations, we set TATB as reference. For each element and absorption edge, the total XRS spectra were constructed by averaging over the number of elements of the corresponding edge and, over the momentum transfer vector parallel to each of the three Cartesian coordinates.

To obtain the excited state electron densities, exciton (photoelectron—core-hole pair) wavefunctions were calculated using an adaptation of the generalized minimal residual (GMRES) algorithm implemented in OCEAN[13]. Usually, for high-quality plots of the electron densities, a finer sampling of the unit cell is required than for calculating x-ray spectra. For all our systems, a real-space uniform sampling of 0.2 a.u. was used for plotting the excited state electron densities.

Data processing

The fitting procedure employed to extract the x-ray Raman scattering (XRS) spectrum is outlined as follows. The model fit is composed by two components. First, an exponential decay function, accounting for the high-energy tail of the valence electron contribution, was fitted to the total energy-loss spectrum for energies below 282 eV (396 eV) the carbon (nitrogen) 1s edge. Second, for energies above 330 eV (430 eV) for the carbon (nitrogen) edge, in addition to the exponential decay function, FEFF calculated atomic backgrounds $S_0(q,\omega)$[14] were added to approximate the high-energy tail of the whole excitation spectra. Then, the obtained fitted exponential is subtracted from the whole energy-loss spectrum to retrieve the XRS contribution.

For calculating the $S_0(q,\omega)$, a cluster containing the optimized geometry of the respective molecule, namely TATB, HNS or CL-20, was employed. For each absorption edge, spherically averaged NRIXS-EXAFS calculations were performed for a momentum transfer of 1.24 a.u. Hedin-Lundqvist exchange-correlation potential with a self-consistent field radius of 5.5 Å were employed, including angular momentum channels up to $l = 3$ to the basis set. For the excitation matrix, angular momentum channels up to $l = 15$ were included.

References

1. Salahub, D. R. *et al.* Qm/mm calculations with demon2k. *Molecules* **20**, 4780–4812, DOI: 10.3390/molecules20034780 (2015).
2. Calaminici, P., Janetzko, F., Köster, A. M., Mejia-Olvera, R. & Zuniga-Gutierrez, B. Density functional theory optimized basis sets for gradient corrected functionals: 3d transition metal systems. *The J. Chem. Phys.* **126**, 044108, DOI: 10.1063/1.2431643 (2007). https://doi.org/10.1063/1.2431643.
3. Huzinaga, S. Gaussian-type functions for polyatomic systems. i. *The J. Chem. Phys.* **42**, 1293–1302, DOI: 10.1063/1.1696113 (1965). https://doi.org/10.1063/1.1696113.
4. Fleischer, U. *et al.* *Deuterium and Shift Calculation* (Springer Berlin, Heidelberg, Heidelberg, Germany, 1991), 1 edn.
5. Triguero, L., Pettersson, L. G. M. & Ågren, H. Calculations of near-edge x-ray-absorption spectra of gas-phase and chemisorbed molecules by means of density-functional and transition-potential theory. *Phys. Rev. B* **58**, 8097–8110, DOI: 10.1103/PhysRevB.58.8097 (1998).
6. Takahashi, O. & Pettersson, L. G. M. Functional dependence of core-excitation energies. *The J. Chem. Phys.* **121**, 10339–10345, DOI: 10.1063/1.1809610 (2004). https://doi.org/10.1063/1.1809610.
7. Gilmore, K. *et al.* Efficient implementation of core-excitation bethe–salpeter equation calculations. *Comput. Phys. Commun.* **197**, 109–117, DOI: https://doi.org/10.1016/j.cpc.2015.08.014 (2015).
8. Vinson, J., Rehr, J. J., Kas, J. J. & Shirley, E. L. Bethe-salpeter equation calculations of core excitation spectra. *Phys. Rev. B* **83**, 115106, DOI: 10.1103/PhysRevB.83.115106 (2011).
9. Giannozzi, P. *et al.* QUANTUM ESPRESSO: a modular and open-source software project for quantum simulations of materials. *J. Physics: Condens. Matter* **21**, 395502, DOI: 10.1088/0953-8984/21/39/395502 (2009).
10. van Setten, M. *et al.* The pseudodojo: Training and grading a 85 element optimized norm-conserving pseudopotential table. *Comput. Phys. Commun.* **226**, 39–54, DOI: https://doi.org/10.1016/j.cpc.2018.01.012 (2018).
11. Hamann, D. R. Optimized norm-conserving vanderbilt pseudopotentials. *Phys. Rev. B* **88**, 085117, DOI: 10.1103/PhysRevB.88.085117 (2013).
12. https://github.com/jtv3/oncvpsp.
13. Vinson, J., Jach, T., Müller, M., Unterumsberger, R. & Beckhoff, B. Quasiparticle lifetime broadening in resonant x-ray scattering of nh_4no_3. *Phys. Rev. B* **94**, 035163, DOI: 10.1103/PhysRevB.94.035163 (2016).
14. Soininen, J. A., Ankudinov, A. L. & Rehr, J. J. Inelastic scattering from core electrons: A multiple scattering approach. *Phys. Rev. B* **72**, 045136, DOI: 10.1103/PhysRevB.72.045136 (2005).
